# New Appliances and Things Medical

**Published:** 1902-07-05

**Authors:** 


### NEW APPLIANCES AND THINGS MEDICAL.

**[We shall be glad to receive at our Office, 28 & 29 Southampton Street, Strand, London, W.C., from the manufacturers, specimens of all new preparations and appliances which may be brought out from time to time.]**

### MALLOWEEN PREPARATIONS.

(HOLME PREPARATIONS COMPANY, 34 AND 35 NORFOLK STREET, STRAND, LONDON, W.C.)

THE Malloween preparations, namely the lotion and the toilet powder, appear from our examination to be pure and safe applications for general and domestic use. Malloween is a skilfully combined fluid in which emollients and antiseptics are united with an extract of marsh mallow. A combination of this kind is well calculated to improve and maintain the health of the skin, partly because it tends to prevent fermentation and decomposition of the retained secretions in the pores of the skin, and partly because it encourages a healthy action of the glands. It induces a cooling and refreshing sensation and is extremely soothing to irritable skins. The powder combines the properties which are commended by dermatologists; it is finely divided and non-irritating, contains no poisonous substance, and has no tendency to clog the pores of the skin.

### FRIEDRICHSHALL MINERAL WATER.

(PROPRIETORS: C. OPPEL AND CO. LONDON OFFICE, 10 AND 12 MILTON STREET, LONDON, E.C.)

THIS mineral water belongs to the class of the muriated-sulphated waters. It contains a large percentage of chloride of soda, and of chloride of magnesium, a percentage which is sufficient to modify the action of the sulphates of sodium and magnesium, to which salts it owes its aperient action. As a mineral water for constant use in the treatment of constipation, and dyspepsia allied with constipation, Friedrickshall has the supreme advantage that it neither irritates the gastric mucous membrane nor loses its efficacy by continued use. It must be regarded as one of the safest and most reliable mineral waters for the cure of inflammatory disorders of the bowel, cholilithiasis, and gout, and as an invaluable prophylactic against appendicitis and gravel.

### DOLLE'S AROMATIC IRON MILK.

(BRITISH IRON MILK SYNDICATE, LIMITED, SAVOY HOUSE, 115 AND 116 STRAND, LONDON, W.C.)

THE merit of Dolle's Aromatic Iron Milk lies in the fact that an insoluble iron salt, namely pyrophosphate of iron, is held in suspension in a milk-like aromatic vehicle composed largely of spirit glycerine and sugar. It is thus possible to administer this valuable iron preparation in the form of a draught instead of in a pill, thus ensuring more immediate and reliable absorption from the alimentary tract. It is a well-known fact that an exclusive milk diet is a sure and satisfactory means of treating most forms of anæmia, and yet milk itself contains only a very small percentage of combined iron. Dolle's Aromatic Iron Milk also contains only a small percentage of pyrophosphate of iron, and yet practical experience shows that its administration increases the hæmoglobin contents of the blood more rapidly, and thus cures anæmic conditions with greater facility than is the case when more powerful and astringent preparations of iron are employed. Dolle's Aromatic Iron Milk is agreeable to the taste. A most satisfactory tonic for infants or children, it does not disturb digestion or damage the teeth.

